# Cognitive Effect of Levetiracetam in Patients with Alzheimer’s Disease or Mild Cognitive Impairment: A Systematic Review

**DOI:** 10.1016/j.curtheres.2025.100798

**Published:** 2025-05-27

**Authors:** Mohamad Hosein Mohamadi, Amir Bavafa, Sahar Salehi, Mahsa Abedi, Fahimeh Shahabi, Sana Jafarlou, Pirhossein Kolivand, Sajad Sahab-Negah

**Affiliations:** 1 Student Research Committee, Sabzevar University of Medical Sciences, Sabzevar, Iran; 2 Neuroscience Research Center, Mashhad University of Medical Sciences, Mashhad, Iran; 3 Department of Neuroscience, Faculty of Medicine, Mashhad University of Medical Sciences, Mashhad, Iran; 4 Department of Food Nanotechnology, Research Institute of Food Science and Technology, Mashhad, Iran; 5 Institute for Molecular and Clinical Immunology, Medical Faculty, Otto-von-Guericke University, Magdeburg, Germany; 6 Department of Health Economics, School of Medicine, Shahed University, Tehran, Iran; 7 Multiple Sclerosis Research Center, Neuroscience Institute, Tehran University of Medical Sciences, Tehran, Iran

**Keywords:** Alzheimer’s disease, Levetiracetam, Mild cognitive impairment

## Abstract

**Background:**

Various therapeutic interventions have been investigated for cognitive impairment, a common problem in Alzheimer’s disease (AD). Levetiracetam (LEV), an antiepileptic drug, has been shown to alleviate cognitive impairment.

**Objective:**

The present systematic review aimed to evaluate the cognitive effects of LEV in patients with AD or mild cognitive impairment (MCI).

**Methods:**

We searched PubMed/MEDLINE, Scopus, Web of Sciences, and Embase databases for all studies on LEV and cognitive impairment. After multistep screening, we identified qualified interventional studies and performed further data extraction. We reviewed the methodological diversity across the studies and assessed the quality of each study using the critical appraisal of the Joanna Briggs Institute checklist (the risk of bias assessment).

**Results:**

Of the 1091 publications, only 5 articles were qualified for review. All studies enrolled patients with AD or MCI, and at least 1 arm of the trial involved LEV therapy. Four of 5 studies reported significant cognitive improvement in patients with AD or MCI after the LEV trials, whereas 1 study found no significant change in cognitive status. The risk of bias assessment revealed that 4 studies had a low risk of bias. Among them, 3 showed significant improvement, whereas 1 did not report a significant change in cognitive function.

**Conclusions:**

The efficacy of LEV therapy for cognitive impairment varies across studies owing to different methodologies, dosages, treatment durations, and outcome assessment tools. This study suggests that LEV may exert a beneficial impact on cognitive function in patients with AD or MCI. However, a quantitative comparison or meta-analysis is essential to draw definitive conclusions about the cognitive effects of LEV in AD and MCI.

## Introduction

Concerns regarding cognitive decline have been growing among the aging population because it can affect attention, executive function, and working memory.^1^, ^2^, ^3^ This condition can range from mild cognitive impairment (MCI) to late-stage Alzheimer’s disease (AD), reducing quality of life and predisposing to neurologic and psychiatric disorders.^4^ The number of cognitive disorders is estimated to triple by 2050, with dementia affecting 152 million people worldwide.^5^^,^^6^

Cognitive impairment is characterized by several key factors, including hyperactive acetylcholinesterase and N-methyl-D-aspartate receptors, as well as the buildup of amyloid β and disruption of synaptic function.^7^^,^^8^ The cognitive deficits are attributed to compromised long-term potentiation, disrupted neuronal calcium signaling, and altered neural plasticity.^9^ Although existing treatments target these aspects, they can only slow the disease’s advancement rather than reverse cognitive decline. Consequently, it is crucial to investigate new and alternative therapeutic strategies.^10^

Levetiracetam (LEV) is an antiepileptic drug that exerts its therapeutic effects by binding to glycoprotein 2A of the synaptic vesicle, regulating the release of neurotransmitters, including γ-aminobutyric acid (GABA), glutamate, dopamine, and serotonin.^11^ Its unique mechanism of action suggests that it may exert positive effects on cognition. In preclinical models of AD, LEV has been shown to improve cognitive characteristics by reducing amyloid β accumulation and enhancing synaptic function.^12^, ^13^, ^14^ Additionally, it was observed that LEV can alleviate cognitive decline in AD animal models by improving neuronal network dysfunction.^15^ These findings underscore the need for clinical studies to determine whether LEV can enhance cognitive function in humans, although this translation remains challenging due to the limitations of animal models and the complex pathophysiology of cognitive impairment in humans.^16^ Therefore, clinical trials are necessary to determine the efficacy and safety profile of LEV in patients with MCI or AD.

Although literature exists on this topic, a comprehensive and current systematic review on the effect of LEV treatment in cognitive function in patients with AD and MCI is scarce. This research aims to review the latest evidence on the cognitive effects of LEV in interventional studies with human patients with AD or MCI. The findings of this study could offer significant insights into the potential applications of LEV in AD and MCI.

## Methods

The present systematic review was conducted according to the Preferred Reporting Items for Systematic Reviews and Meta-Analysis statements.

### Search strategy

We searched PubMed/MEDLINE, Web of Science, Scopus, and Embase databases. We also searched the ClinicalTrials.gov registry for relevant published findings. The search was performed on January 1, 2023, with no specific time restrictions. To develop the search criteria, we used appropriate terms, including “Alzheimer’s disease,” “AD,” “cognitive impairment,” “mild cognitive impairment,” “MCI,” “dementia,” “levetiracetam,” “Keppra,” and “AGB101.” We specifically searched for these terms and structures in the titles and abstracts of research studies. We did not restrict the studies’ language, location, or settings. Keppra is the brand name for LEV, and AGB101 is a low-dose formulation of LEV, the active ingredient in Keppra.^17^^,^^18^

### Inclusion and exclusion criteria

We searched for studies that investigated the use of LEV therapy for cognitive improvement. All search results from different databases were collected using EndNote X9 software (Thomson Reuters, San Francisco, California), and duplicate studies were removed. We assessed the studies based on their titles, abstracts, and full texts. After a 3-stage screening process, eligible studies were selected. We included interventional studies, including clinical trials with or without randomization, blinding, and crossover designs. Observational studies were not included in this systematic review. We focused on clinical trials on AD or MCI. Some of them reported that their populations included patients with AD with comorbid epilepsy. These studies were not excluded and remained part of the systematic review. We only excluded the studies that involved epilepsy patients without AD or MCI.

The Diagnostic and Statistical Manual of Mental Disorders, Fifth Edition, defines 6 neurocognitive domains including: 1) perceptual-motor function, 2) language, 3) executive function, 4) complex attention, 5) social cognition, 6) learning and memory^19^ To be included, the studies had to evaluate at least 1 of these cognitive domains. The cognitive domains and their corresponding evaluation methods were reviewed based on established principles of cognitive testing, as presented in Table 1.^20^, ^21^, ^22^, ^23^, ^24^, ^25^, ^26^, ^27^, ^28^, ^29^, ^30^, ^31^, ^32^, ^33^, ^34^ We excluded articles in the following categories: review articles, meta-analyses, duplicate publications by the same researchers, congress abstracts, animal studies, in vitro studies, and observational studies.

**Table 1:** Cognitive assessment in included studies.

Cognitive test	Cognitive domains assessed	Principle of the test	Reference
Free Recall Test	Episodic memory, verbal learning	Measures the ability to recall words or items from a presented list after a delay.	(20)
TMT A+B)	Processing speed (A), executive function, cognitive flexibility (B)	TMT-A requires connecting numbers in order; TMT-B requires alternating between numbers and letters.	(21)
Phonemic Fluency Test	Executive function, verbal fluency	Requires generating as many words as possible starting with a given letter in a limited time.	(22,23)
Boston Naming Test	Language, lexical retrieval	Measures naming ability by asking participants to identify pictured objects.	(24)
Free and Cued Selective Reminding Test	Episodic memory, verbal learning	Evaluates memory recall with both free recall and cued recall conditions.	(25,26)
Clock Drawing Test	Visuospatial ability, executive function	Requires drawing a clock with a specific time, testing spatial organization and planning.	(27,28)
Mini-Mental State Examination	Orientation, memory, attention, language, visuospatial skills	Brief cognitive screening test with tasks like recall, calculation, and language.	(29-31)
Alzheimer’s Disease Assessment Scale-Cognitive	Memory, language, praxis, executive function	Comprehensive cognitive assessment used in AD clinical trials, testing multiple domains.	(32)
NIH Examiner Test	Executive function, working memory, attention	Assesses cognitive control and reasoning, often including computerized tasks.	(33)
Stroop Test	Executive function, attention, cognitive flexibility	Measures response inhibition by requiring naming ink colors of incongruent words.	(34)

TMT, Trail Making Test.

### Data collection

At least 2 independent authors extracted data from each article. A third author resolved any discrepancies or inconsistencies between the investigators. The collected data are organized and presented in Table 2.^35^, ^36^, ^37^, ^38^, ^39^

**Table 2:** General characteristics of included studies.

Study	Country	Randomization and blinding	Study population	ClinicalTrials.gov registration	References
1	USA	Nonrandomized, no blinding	AD and MCI with epileptic seizures	Not registered	(35)
2	Italy	Randomized, double-blind	AD with epileptic seizures	Not registered	(36)
3	USA	Randomized, double-blind	AD	Registered (NCT01554683)	(37)
4	USA	Randomized, double-blind	AD	Registered (NCT02002819)	(38)
5	USA	Randomized, double-blind	MCI	Not registered	(39)


								
Study	Study groups	Sample size	Dropout	Gender	Mean age	Interventions and dosage	Duration of treatment	References
1	Treatment group (n = 24)	Entry: 24 Completed trial: 16	• Lost to follow-up due to conflict with caregiver (n = 3), • Discontinue the drug due to fatigue as a side effect (n = 4), • Died from nondrug-related causes (n = 1)	M = 13 F = 11	69.9	Levetiracetam (initial dose: 500 mg daily, final dose: 500–300 mg daily)	12 wks	(35)
2	Control (n = 68) Levetiracetam(n = 38), phenobarbital(n = 28), lamotrigine(n = 29)	Entry: 163 Completed trial: 151	• From the levetiracetam group, 3 patients were lost to follow-up due to noncompliance (with no adverse events)	M = 71 F = 92	71.89 (total participants) 72.02 (LEV group)	• Levetiracetam (mean dosage = 956 mg/d-initial dosage: 500 mg/d) • Lamotrigine (mean dosage = 57/5 mg/d-initial dosage: 25 mg/d) • Phenobarbital (mean dosage = 90 mg/d-initial dosage: 50 mg/d) • Each group received 1 drug	12 mos	(36)
3	Group A Group B Group C	Entry: 12 Completed trial: 7	• Claustrophobia inside the MRI scanner (n = 2), • Desire not to perform the cognitive test (n = 1), • Muscle artifacts on EEG and inadequate awake EEG (n = 2)	M = NR F = NR	NR	• Levetiracetam (first dose: 2.5 mg/kg, second dose: 7.5 mg/kg) • Placebo • Totally 3 doses ([one 2.5 Lev], [one 7.5 Lev], [one placebo] doses)	Three doses (3 sessions that are separated by 1 wk)	(37)
4	Group A (n = 17) Group B (n = 17)	Entry: 34 Completed trial: 28	• Subclinical epileptiform activity as an exclusion criterion (n = 2) • Not able to perform Stroop test (n = 2) • Receiving antiseizure medication for any reason (n = NR)	M = 13 F = 21	Overall mean (62.35)	• Levetiracetam (250 mg, capsule, daily) • Placebo	Two phases Phase I: 4 wks Phase II: 4 wks	(38)
5	aMCI (n = 23), healthy control (n = 22)	Entry: 45 Completed trial: 34	• Not completing study protocol (inability to complete MRI sessions and noncompliance) (n = 11)	M = 15 F = 20	71.15 (total) 72.9 (aMCI)	• Levetiracetam (250 mg, capsule, daily) • Placebo	Two phases Phase I: 2 wks Phase II: 2 wks	(39)

AD = Alzheimer’s disease; aMCI = amnestic mild cognitive impairment; F = female; M = male; MCI = mild cognitive impairment; MRI = magnetic resonance imaging; NR = not reported.

### Quality assessment

The quality of all included studies was assessed using the Joanna Briggs Institute critical appraisal checklist, specially designed for clinical trial studies. This checklist assesses bias at various levels, including selection and allocation, intervention, outcome measurement, participant retention, statistical validity, and study design. This checklist consists of 13 questions with response options of “yes,” “no,” “unclear,” or “not applicable.” The quality of the studies was determined based on the total number of “yes” answers, where ≥70% was considered high quality, 50% to 69% moderate quality, and <50% low quality.^40^, ^41^, ^42^

## Results

### Study characteristics

**Figure 1:**
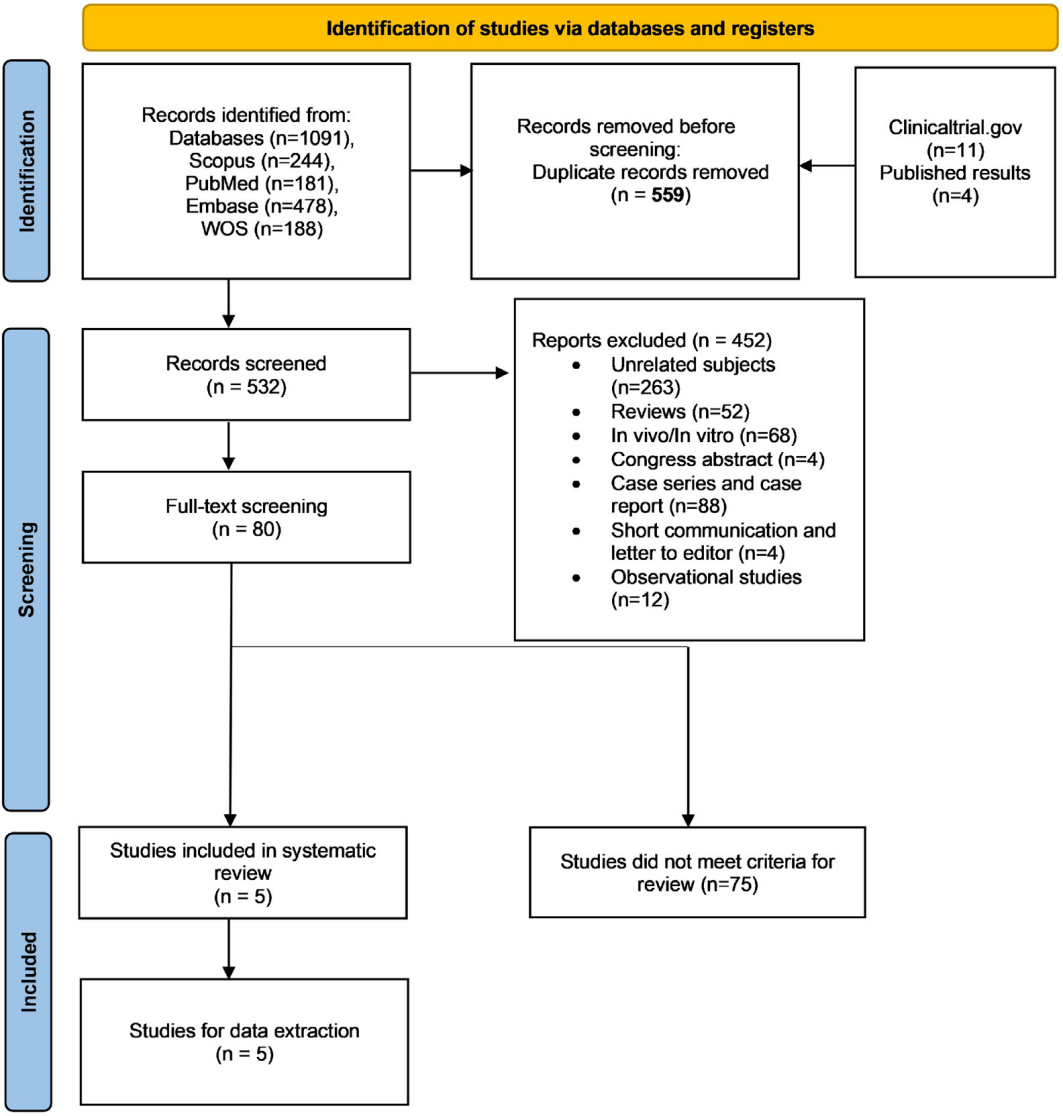
Flow diagram of study screening and selection process.

According to the flow diagram (Figure 1), 1091 papers were initially retrieved from 5 databases, and 11 results were collected from the ClinicalTrials.gov registry. After removing duplicates, 532 studies were screened based on their titles and abstracts. Only 80 studies met the inclusion criteria and underwent further evaluation through full-text screening. Finally, 5 articles qualified for data extraction and were included in the systematic review.

The included studies employed diverse methodologies and study designs. Of the 5 studies, 3 were randomized crossover trials, 1 was a randomized controlled trial, and 1 was an open-label trial. All were randomized and double-blinded, except for 1 study which was open-label trial. Three studies were previously registered at ClinicalTrials.gov. The details of the included studies are presented in Table 2.

### Population

The study population consisted of patients with MCI or AD. Given that LEV is an anticonvulsant drug, 2 studies reported that their populations included patients with AD with comorbid epilepsy.^35^^,^^36^ They were included in this review because their inclusion criteria required the presence of either AD or MCI. In screening, we identified a few studies that have focused on patients with epilepsy without AD or MCI.^43^^,^^44^ Because these studies included epilepsy patients without MCI or AD, they did not meet the inclusion criteria and were excluded.

Four studies reported study groups with randomization and double-blinding, allowing for a comparison between the LEV and control groups (Table 2). However, 1 study conducted a single-arm open-label study of LEV without any control group.^35^ Consequently, statistical between-group analysis could not be performed.^35^ One study mentioned that only 7 patients completed the trial, thus posing a challenge because of the small sample size.^37^ The limited sample size may have affected the generalizability of the findings. Therefore, a larger population is recommended to validate the LEV effects. By reviewing the databases, we found only 5 LEV studies on MCI and AD.

### Treatment

First, we evaluated the route of LEV administration. The included articles predominantly reported using LEV in capsule or tablet forms,^35^^,^^36^^,^^38^^,^^39^^,^^45^ which is commonly used in outpatient settings. However, Musaeus CS et al used an intravenous LEV injection.^37^ The review of the findings revealed that 4 of 5 studies reported significant cognitive changes after LEV treatment (Table 2). The study, which reported no significant changes, was classified as low quality due to the lack of randomization, blinding, and a control group, as well as concerns about the interpretation of the statistical analyses. The study reported various doses of LEV over 3 months and then compared cognitive status before and after treatment. The included studies assessed various cognitive domains, as presented in Table 3.^35^, ^36^, ^37^, ^38^, ^39^ Two studies focused on AD,^36^^,^^38^ 1 study focused on MCI,^39^ and another study included both AD and MCI.^35^ They used varying dosages and durations of LEV treatment. Both daily doses of 250 mg and 500 mg were shown to be effective in improving cognitive domains. A 250 mg daily dose of LEV significantly improved spatial memory and executive function,^38^ whereas a 500 mg daily dose of LEV significantly enhanced global cognitive function (measured by Alzheimer Disease Assessment Scale-Cognitive Subscale [ADAS-Cog] and Mini-Mental State Examination [MMSE]).^36^ The duration of treatment influenced the cognitive effects of LEV. Various durations of treatment were reported by the included studies. Studies with treatment durations of 2 weeks,^39^ 4 weeks,^38^ 12 weeks,^35^ and 12 months^36^ reported significant results, whereas a clinical trial with 3 sessions of LEV administration did not report significant results^37^ (Table 2). These findings indicate that the duration of treatment may influence the LEV cognitive effects. For future research, it will be crucial for investigators to conduct LEV trials in individuals with AD or MCI to determine the optimal dosage and duration of treatment for achieving effective outcomes.

**Table 3:** Data of the included studies: outcome, assessment, adverse events, and analysis.

Study	Cognitive domain assessed	Cognitive test	Cognitive change	Analysis protocol	References
1	• Global cognitive function	• MMSE • ADAS-cog	• MMSE (+2.2 improved, sig), • ADAS-cog (−4.3 improved, **sig**)	Intention-to-treat analysis	(35)
2	• Global cognitive function	• MMSE • ADAS-cog	• MMSE (+0/23 improved, **sig**) • ADAS-Cog (−0/023 improved, **sig**)	Intention-to-treat analysis	(36)
3	• Executive function • Declarative memory • Overall cognitive performance	• Free recall test • Trail Making Test Parts A+B • Phonemic fluency Test, Boston Naming Test • 15-item version, clock drawing	• Free recall test (NS) • Semantic fluency score (NS) • Trail-making test (NS) • MoCA (NS)	Per-protocol analysis	(37)
4	• Executive function • Executive functions • Global cognitive • Spatial memory • Executive function	• NIH-EXAMINER test • Stroop test • ADAS-cog • VRLT	• NIH-EXAMINER test (NS) • Stroop test (NS) • ADAS-cog (NS) • Spatial memory (**sig**) • Executive function (**sig**)	As-treated analysis	(38)
5	• Verbal memory	• Memory performance	• Memory performance (**sig**)	Per-protocol analysis	(39)

Study	Adverse events related to levetiracetam	Other treatments		ClinicalTrials.gov registration	References

1	Fatigue (n = 5), dizziness (n = 1)	• Cholinesterase inhibitor (n = 5), • Memantine (n = 4), • Cholinesterase inhibitor + memantine (n = 2)		Not registered	(35)
2	Somnolence (n = 2), Dizziness (n = 1), Headache (n = 1), Asthenia (n = 2)	• Cholinesterase inhibitor(as standard treatment for all groups)		Not registered	(36)
3	NR	• Cholinesterase inhibitor(as standard treatment for all patients)Additionally, 2 patients received mirtazapine or paroxetine		Registered	(37)
4	Dizziness (n = 2), GI discomfort (n = 3), headache (n = 5), irritability (n = 2) nausea (n = 2), tiredness (n = 2) vivid dreams (n = 2)	• Cholinesterase inhibitor(as standard treatment for all groups)		Registered	(38)
5	NR	NR		Not registered	(39)

ADAS-Cog = Alzheimer’s Disease Assessment Scale-cognition sub-scale (ADAS-Cog); GI = gastrointestinal; MMSE = Mini-Mental State Examination; MoCA: Montreal Cognitive Assessment; NIH-EXAMINER = National Institutes of Health Executive Abilities: Measures and Instruments for Neurobehavioral Evaluation and Research; NR = not reported; VRLT = virtual route learning test. NS: not significant; sig: significant.

### Cognitive assessment

Cognitive assessment involves evaluating multiple cognitive domains after a defined period of LEV treatment. The Diagnostic and Statistical Manual of Mental Disorders, Fifth Edition, defines 6 neurocognitive domains and their subdomains: language, executive function, learning, and memory. Researchers have also assessed other aspects of cognition, such as associative and declarative memory. Various assessment tools have been used, including the ADAS-Cog and MMSE, although no standardized tool has been universally adopted.^35^^,^^36^^,^^38^ Four of the 5 studies included in this review reported significant cognitive improvements after LEV treatment (Table 3).^35^^,^^36^ The comparison of their cognitive improvement was not feasible due to the use of different cognitive assessment tools. The cognitive tests and their principles are presented in Table 1. To achieve better results, future investigations should focus on developing a single applicable tool for cognitive assessment, enabling meta-analyses and statistical conclusions.

### Risk of bias assessment

Four studies were controlled clinical trials, and 1 was a single-arm open-label trial.^35^ First, we reviewed the single-arm trial which was classified as low quality due to the absence of randomization and blinding in its study design.^35^ They reported that no concealment was implemented, and the participants did not match their baseline characteristics.^35^ In that study, the researchers compared the effect of LEV before and after the follow-up periods, which was statistically appropriate. Still, the outcome could not be attributed to LEV treatment. However, there were 4 good-quality studies with a low risk of bias.^36^, ^37^, ^38^, ^39^ Among these, 3 studies reported a significant effect of LEV treatment in patients with AD. Two of them identified a significant change in the primary outcome,^36^^,^^39^ and another study found significant change in the secondary cognitive outcomes, such as spatial memory and executive function.^38^ By reviewing, 3 studies were identified as crossover trials. All crossover studies considered an adequate washout period and provided detailed descriptions of the follow-up events. They also reported instances of participant dropouts (reasons included inability to complete the magnetic resonance imaging session and failure to complete the study protocol for any reason), missed doses, and noncompliance. Three studies reported adverse events associated with LEV.^35^^,^^36^^,^^38^ Study design is one of the important components of the appraisal tool that was evaluated in this review. Two studies had less-than-ideal design: (1) an open-label trial^35^ and (2) a trial involving 3 sessions of drug injection.^37^ The other 3 studies presented methodologically appropriate designs. Studies with treatment durations of 2 weeks,^39^ 4 weeks,^38^ 12 weeks,^35^ and 12 months^36^ reported significant results, whereas a clinical trial with 3 sessions of LEV administration did not report significant results.^37^ It seemed that the duration of treatment could be a crucial factor in the effectiveness of LEV for cognitive impairment.^36^ Furthermore, conflict of interest is a key component of the risk of bias assessment that was evaluated. Two of the 5 studies disclosed a conflict of interest,^38^^,^^39^ whereas the remaining 3 reported no competing interests (Table 4).^35^, ^36^, ^37^, ^38^, ^39^

**Table 4:** Risk of bias assessment: JBI appraisal for controlled clinical trial.

Study	Selection and allocation bias			Intervention bias			Outcome level bias			Participant retention bias	Statistical validity		Design	Score (yes)
	Randomization	Concealment	Similar baseline	Blindness	Blinded intervention treatment	Identical treatment groups	Blinded outcome assessor	Similar outcome measure	Reliable outcome measure	Follow-up description	ITT analysis	Appropriate statistical analysis	Appropriate design	
Study 1 (35)	No	No	Unclear	No	No	No	No	Yes	Yes	Yes	Yes	Yes	No	5
Study 2 (36)	Yes	Yes	Yes	Yes	Yes	Yes	Yes	Yes	Yes	Yes	Yes	Yes	Yes	13
Study 3 (37)	Yes	Yes	Yes	Yes	Yes	Yes	Unclear	Yes	Unclear	Yes	No	Yes	Yes	10
Study 4 (38)	Yes	Yes	Yes	Yes	Yes	Yes	Yes	Yes	Yes	Yes	No	Yes	Yes	12
Study 5 (39)	Yes	Yes	Yes	Yes	Yes	Unclear	Yes	Yes	Unclear	Yes	No	Yes	Yes	10

ITT = intention-to-treat.

## Discussion

This study aimed to investigate the effects of LEV on the cognitive abilities of patients diagnosed with MCI or AD. This is a crucial area of research because previous studies have reported effective therapeutic options only in AD,^46^ but there is a lack of effective treatments for MCI.^47^ Moreover, preclinical studies support the hypothesis that LEV enhances cognitive performance.^15^^,^^48^^,^^49^ Therefore, it is necessary to review the previous evidence to explore the clinical findings in this field.

The results of this systematic review suggest that LEV may have a positive effect on cognitive function in patients with MCI or AD. Supporting this, Lippa et al^35^ conducted a nonrandomized, open-label clinical trial which reported improvements in MMSE and the ADAS-Cog after 12 weeks of LEV use. It is notable that the risk of bias assessment for this study revealed a low-quality score. Another study supported the LEV-associated cognitive improvement after 3- and 6-month follow-up.^36^ In that study, patients who were seizure-free reported steady improvement from baseline to the 3-month to the 6-month follow-up, but patients with seizures reported an initial improvement that plateaued between 3-month and 6-month of LEV use.^36^^,^^44^ These 2 studies assessed global cognitive function using MMSE and ADAS-Cog in AD.^35^^,^^36^ Another randomized controlled trial employed a crossover design and reported significant cognitive changes after LEV treatment in patients with AD.^38^ The investigators assessed executive function as primary outcome and spatial memory and executive function as secondary outcomes. The primary outcomes were measured by the National Institutes of Health Executive Abilities: Measures and Instruments for Neurobehavioral Evaluation and Research. Although the secondary outcomes were measured by the Stroop Color and Word Test (Stroop) interference naming subscale and ADAS-Cog. They reported that LEV did not improve primary outcome, but in further analysis, LEV improved spatial memory performance and executive function tasks in patients with AD and epileptiform activity.^38^ Another crossover trial reported significant improvement in task-related memory after LEV treatment in patients with MCI.^39^ They found that LEV reduced hippocampal hyperactivity, leading to improved cognition in patients with MCI. They evaluated how LEV influenced the CA1, dentate gyrus/CA3 (DG/CA3), and subiculum subregions of the hippocampus using functional magnetic resonance imaging.^39^ Then, they found that LEV improved memory via regulating the hippocampal activation in patients with MCI.^39^

Among the included studies, 2 noted that patients with AD and MCI also had epileptic seizures.^35^^,^^36^ Both studies reported significant cognitive improvement after LEV treatment. Notably, another study found that LEV enhanced cognitive function in patients with AD only in the presence of epileptiform activity.^38^ These findings suggest that LEV may exert positive effects on cognitive function in AD or MCI, particularly in the presence of epilepsy as a comorbidity. Further research is needed to determine the specific conditions under which LEV exerts its effects.

Besides efficacy, the safety of treatment is a major concern that should be addressed. Considering that cognitive disorders mainly occur in old age,^50^ the use of safe therapeutic agents becomes a critical issue. Three studies reported adverse events associated with LEV.^35^^,^^36^^,^^38^ Dizziness, fatigue, and headache were the most common adverse effects. Only 4 patients discontinued the LEV trial due to fatigue.^23^ No life-threatening events were reported in association with LEV in the included studies. Beyond the included studies, some severe adverse events have been reported for LEV, including thrombocytopenia, anemia, angioedema, acute liver failure, acute renal failure, rhabdomyolysis, eosinophilia, pneumonia, Stevens–Johnson syndrome.^51^ Additionally, Cumbo et al^36^ noted that LEV exhibited fewer side effects than lamotrigine and phenobarbital. Although 3 studies reported some side effects, they were not critical, allowing patients to continue the trial. LEV does not cause considerable changes in blood laboratory factors.^52^ It seemed that LEV treatment was well-tolerated and identified as a safe agent for patients with AD and MCI. Other studies beyond those included in our review have noted that LEV may cause mild side effects, including psychiatric symptoms. They were not serious, life-threatening, or intolerable events.^53^^,^^54^ Considering the minimal side effects, good tolerability, and potential benefits of LEV, it may be a viable therapeutic option for the elderly with MCI or AD.

It is essential to evaluate the other medications that patients are receiving during the trial, in addition to LEV. Many of the included studies mentioned cholinesterase inhibitors as standard treatment of patients with AD, but they did not specify which cholinesterase inhibitor was used.^35^, ^36^, ^37^, ^38^ Because all study groups were similar in terms of cholinesterase inhibitor use, this factor was unlikely to have affected the results.

The study design of each included study was evaluated as part of the risk of bias assessment. Musaeus et al^37^ conducted a double-blinded crossover study, which is appropriate in terms of statistical conclusion validity, and mentioned appropriate washout periods between treatments. Additionally, Vossel et al^38^ conducted a double-blinded cross-over randomized clinical trial. The study design was appropriate because the proper washout periods were considered, and the intervention did not produce a long-term effect. In addition, the statistical conclusion was valid. Another included study was a randomized controlled trial with LEV, lamotrigine, and phenobarbital treatment.^36^ The final comparison and analysis were made between the LEV group and each of the other treatments and control groups. The validity of the statistical conclusion and analysis was assessed. The study design was appropriate to conclude. Lippa et al^35^ evaluated the 12-week effect of LEV in a single-arm trial. The comparison was not appropriate due to the lack of a control group. The study design was not appropriate based on the Joanna Briggs Institute appraisal tool. In general, the conclusion validity was appropriate for 4 of the 5 included studies, which was favorable for drawing a conclusion. We systematically reviewed the literature and extracted findings from included studies. However, further research is needed to generate more comprehensive data, which would support the feasibility of conducting a robust meta-analysis.

In this review, the statistical analysis and methods used by the included studies were assessed. The statistical power and violation of assumptions of statistical tests are 2 important issues that were checked across the included studies. The level of significance was set at 0.05 for all tests that indicated appropriate statistical power. Additionally, all studies used appropriate statistical methods regarding the characteristics of data and objectives of the analysis. Lippa et al^35^ used the Mann–Whitney test to compare the cognitive function between baseline and 12 weeks in a single-arm trial with no control group. Although the cognitive change could not be attributed to LEV treatment, statistical tests were appropriate regarding the nature of their data and study design. However, caution should be taken when interpreting Lippa et al's^35^ study due to its low score in the risk of bias assessment.

This review has several limitations. First, the findings require further validation through additional research. Second, the cognitive tests employed may lack standardization, potentially limiting the comparability of outcomes with other studies. Finally, the study design and group matching processes, including randomization and control group homogeneity, could be improved to enhance the reliability of the findings.

To address these limitations and advance the field, several recommendations are proposed. Standardized cognitive assessments, including uniform tests of executive function, memory, and attention, should be employed to ensure consistency and facilitate cross-study comparisons. Optimizing study designs through appropriate group matching, randomization, and the use of homogeneous control groups would strengthen methodological rigor and enhance the reliability of results. Notably, a meta-analysis is essential to draw definitive conclusions about the cognitive effects of LEV in AD and MCI. However, due to the limited number of studies and methodological heterogeneity, conducting a meta-analysis was not feasible in this review. Further efforts would help elucidate the effects of LEV on AD and MCI. This study suggests that LEV may positively influence cognitive function in patients with AD or MCI. However, further research is required to confirm these findings and determine the specific conditions under which LEV exerts its effects.

## Funding

There is no funding for the present study.

## Declaration of Generative AI and AI-Assisted Technologies in the Writing Process

During the preparation of this work, the authors used ChatGPT in order to enhance the fluency of the sentences. After using this tool/service, the authors reviewed and edited the content as needed and take full responsibility for the content of the publication.

## Ethics Approval and Consent to Participate

Ethical approval was unnecessary for this research, as it utilized data from previously published studies.

## Consent for Publication

All authors have read and approved the final version of the manuscript.

## CRediT authorship contribution statement

**Mohamad Hosein Mohamadi:** Conceptualization, Methodology, Investigation, Writing – original draft, Writing – review & editing. **Amir Bavafa:** Methodology, Writing – original draft, Writing – review & editing, Investigation. **Sahar Salehi:** Methodology, Writing – original draft, Writing – review & editing. **Mahsa Abedi:** Methodology, Writing – original draft. **Fahimeh Shahabi:** Methodology, Writing – original draft, Writing – review & editing. **Sana Jafarlou:** Methodology, Writing – original draft, Writing – review & editing. **Pirhossein Kolivand:** Methodology, Writing – review & editing. **Sajad Sahab-Negah:** Conceptualization, Methodology, Investigation, Supervision, Writing – review & editing.

## Declaration of competing interest

All authors approved that there are no interests to declare.

The authors declare that they have no known competing financial interests or personal relationships that could have appeared to influence the work reported in this paper.
